# Shigellosis Among Gay and Bisexual Men: A Qualitative Assessment to Examine Knowledge, Attitudes, and Practices

**DOI:** 10.1097/OLQ.0000000000001220

**Published:** 2020-06-19

**Authors:** Elise Caruso, Eric R. Wright, Ebony Townsend Respress, Steve L. Evener, Kathleen Jacobson, Anna Bowen, Rachel Kachur, Amanda Garcia-Williams

**Affiliations:** From the ∗ Division of Foodborne, Waterborne, and Environmental Diseases, Centers for Disease Control and Prevention; † Department of Sociology; ‡ School of Public Health, Georgia State University; Divisions of § Parasitic Diseases and Malaria; ¶ STD Prevention, Centers for Disease Control and Prevention, Atlanta, GA

## Abstract

**Background:**

Shigellosis is a highly contagious enteric bacterial disease transmitted through the fecal-oral route. It is primarily transmitted through person-to-person contact and via contaminated food and water. Outbreaks of shigellosis among men who have sex with men (MSM) attributed to sexual person-to-person contact have been reported. These outbreaks are of concern because they are often caused by multidrug-resistant strains of *Shigella*. Little is known about shigellosis-related knowledge, attitudes, and practices among gay, bisexual, and other MSM.

**Methods:**

Six focus groups were conducted among self-identified gay or bisexual men in Atlanta, GA, in Fall 2017. Participants were asked about shigellosis-related knowledge, attitudes, and practices. Focus groups were audio recorded, and the transcribed audio was analyzed using inductive and deductive thematic coding.

**Results:**

Among the 24 focus group participants, most perceived that diarrheal illness was caused by contaminated food. Knowledge of shigellosis and *Shigella* was low, with most never having heard of the disease or bacteria. Participants did not perceive shigellosis to be a serious health concern, especially when compared with HIV; however, they did perceive gay and bisexual men to be at risk of *Shigella* infection. Participants reported mixed intentions to change sexual behaviors to prevent shigellosis or talk with sexual partners about diarrhea.

**Conclusions:**

Health communication and education efforts could be used to increase knowledge about shigellosis and shift perceptions about the severity of shigellosis among gay, bisexual, and other MSM. Additional work is needed to identify effective ways to promote shigellosis-related prevention behaviors among gay, bisexual, and other MSM.

## Abstract 2

A qualitative study among gay and bisexual men examining shigellosis-related knowledge, attitudes, and practices revealed low knowledge levels, limited concern about shigellosis, and mixed intention to engage in prevention behaviors.

Shigellosis is an acute diarrheal illness caused by *Shigella* bacteria and is the third most common bacterial enteric disease in the United States, causing an estimated 500,000 cases annually.^1^*Shigella* bacteria are transmitted through the fecal-oral route, and people can become sick if they come into contact with contaminated food, water, objects, hands, or through sexual contact.^2,3^ Most outbreaks of shigellosis in the United States are attributed to person-to-person contact,^4^ as are most sporadic cases of *Shigella* infection in the United States.^5^ Although most shigellosis cases in the United States occur among children younger than 10 years,^6^ other populations at risk include international travelers^5,7^ and men who have sex with men (MSM).^2,8–10^

Outbreaks of shigellosis have been reported among MSM since the early 1970s.^11^ Since that time, cases, clusters, and outbreaks of shigellosis among MSM have been reported in the United States^12,13^ and elsewhere,^8,10,14–16^ with many of these outbreaks believed to be associated with spread of *Shigella* bacteria through sexual contact.^3^ The burden of sporadic shigellosis among MSM in the United States is not well characterized, and the number of clusters or outbreaks of shigellosis among MSM in the United States is also not known. Shigellosis among MSM is of concern because multiple outbreaks and clusters have been caused by drug-resistant strains of *Shigella.*^3,10,12,13,15^ Drug-resistant *Shigella* is an emerging public health threat,^17^ and resistant infections can be more difficult to treat and can result in more severe health outcomes.

Outbreak and other epidemiological investigations among MSM have identified that a range of behaviors cases have engaged in before becoming sick with shigellosis. This includes oral and anal sex, direct oral-anal contact (e.g., anilingus), multiple sex partners, use of drugs or alcohol before or during sex, and finding sex partners through online dating applications or in sex venues like bath houses.^8–10,12–16^ In addition, coinfection with a sexually transmitted infection or with HIV has also been reported among MSM cases of shigellosis.^8,10,13^ Less is known about unique risk factors for shigellosis among MSM, but some that have been identified include being HIV positive and having direct anal contact before illness onset.^9^ Knowledge and awareness of shigellosis among MSM seem to be low. In one study conducted in the United Kingdom among 3646 MSM, only 26% had heard of *Shigella*, 16% were aware that *Shigella* can cause stomach upset and can be spread by food and through sex, and 16.3% were aware that *Shigella* can spread easily.^18^ In addition, in 2 outbreak investigations in the United Kingdom, few MSM had heard of shigellosis before their illness.^8,10^ For this study, we sought to better understand shigellosis-related knowledge, attitudes, and practices (KAPs) among gay and bisexual men, and identify opportunities for health communication and education efforts.

## METHODS

Six focus group discussions were conducted between November and December 2017 in Atlanta, GA. Participants were eligible for the study if they (1) were 18 years or older, (2) were cisgender male, (3) self-identified as gay or bisexual, (4) reported having sex with another man in the past 3 months, and (5) were able to speak and understand English. The term MSM will not be used to refer to participants in this study as all participants in this study self-identified as gay or bisexual.

Focus group participants were recruited using 2 strategies. First, Georgia State University (GSU) team members partnered with local organizations and health care providers serving gay and bisexual men in the greater Atlanta area to distribute an informational flyer describing the focus groups. Second, study staff asked individuals interested in participating in the focus groups to share information about the project within their social networks. Individuals interested in participating in the focus groups were directed by recruitment materials to call project staff, who screened potential participants for eligibility. Eligible individuals were invited to attend 1 of 6 focus group sessions that best fit with their schedules.

Before the start of the focus groups, participants completed a brief demographic questionnaire that contained questions about age, sex, race, ethnicity, education level, and use of mobile dating applications. A semistructured moderator guide was used to facilitate the focus group discussions. The interview guide included questions on health-related help-seeking behaviors, KAPs related to shigellosis, and health education messaging preferences. Focus groups were audio recorded, and field notes were taken to capture nonverbal responses and emergent focus group themes. Audio recordings of the focus groups were transcribed verbatim, and the transcripts were used as the basis of all analyses.

Thematic analysis was used to analyze focus group transcripts, with segments of texts assigned both deductive and inductive codes. A preliminary codebook was developed after one transcript was collectively coded by 4 researchers. All transcripts were then coded by a minimum of 2 coders. Each transcript was coded deductively using the codebook, and new inductive codes were also applied to transcripts during the coding process. All discordant deductive codes were discussed until 100% agreement between coders was achieved. Newly identified inductive codes were also discussed until 100% agreement on code name, definition, and instructions for use was obtained among coders. These newly identified inductive codes were then added to the codebook and used prospectively.

Once all transcripts were coded and all new inductive codes were added to the codebook, all 6 transcripts were recoded a second time. During this second round of coding, a minimum of 2 coders discussed all codes applied in each transcript, and codes were updated to reflect any changes to the codebook made during the first round of coding. All data management, coding, and analyses were completed using qualitative data management software MAXQDA 12.3.5.^19^

Human subjects research approval was obtained from the GSU institutional review board). Informed consent was obtained from all participants, and copies of informed consent documents were provided to all participants. Participants were provided with a $40 Visa gift card as compensation for their time and were compensated even if they chose to withdraw from the focus group during the session. All recruitment and data collection activities were conducted by GSU.

## RESULTS

A total of 24 participants were recruited for the 6 focus groups. Each focus group discussion lasted approximately 1 hour, and the number of participants in each group ranged from 2 to 6. Participants ranged in age from 21 to 59 years, with a mean age of 36 years. More than half of participants (54%; n = 13) were non-Hispanic African American, 38% (n = 9) were non-Hispanic white, and 8% (n = 2) were Hispanic, of any race. Less than half (46%; n = 11) of participants had completed a 4-year college degree, another 25% (n = 6) reported having postgraduate education, and 25% (n = 6) had completed high school or some college (Table 1). All participants (100%; n = 24) reported using smartphone applications to meet sexual partners.

**TABLE 1:**
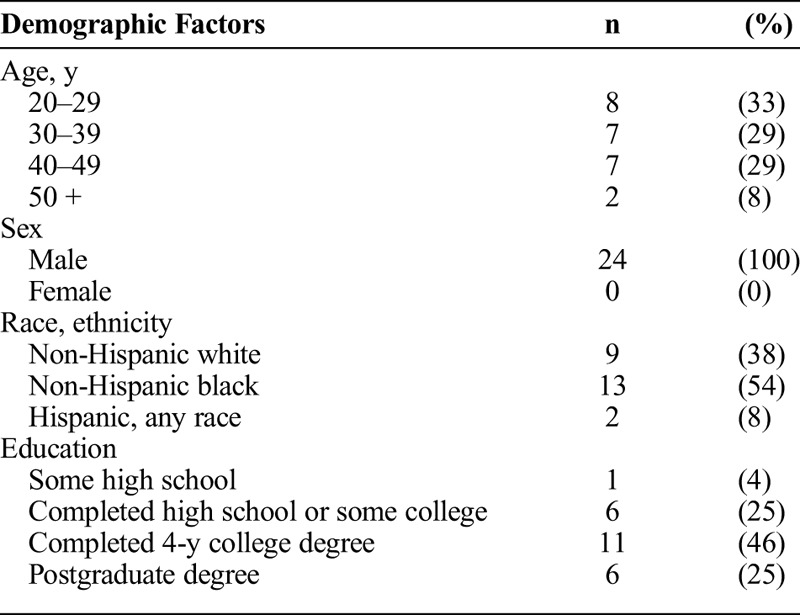
Self-Reported Demographic Characteristics of Focus Group Participants (n = 24)

### Knowledge

Across all participants, knowledge about *Shigella* and shigellosis was limited (Table 2). Most participants had not heard of shigellosis before the focus groups, and several participants reported that when they heard the word *Shigella*, they thought of the disease shingles, suggesting that *Shigella* “sounds something like shingles.” Among the small minority of participants who had heard of *Shigella* or shigellosis before, knowledge was generally limited, with some participants able to describe some basic characteristics of *Shigella* infection, including transmission, contagiousness, and bacterial etiology. For example, one participant said, “I've heard of it, I'm not sure I know what it is, but I've heard of it.” Of the few participants who had heard about *Shigella*, they had either learned about it through social networks like friends or as a result of working or studying within the health care field.

**TABLE 2:**
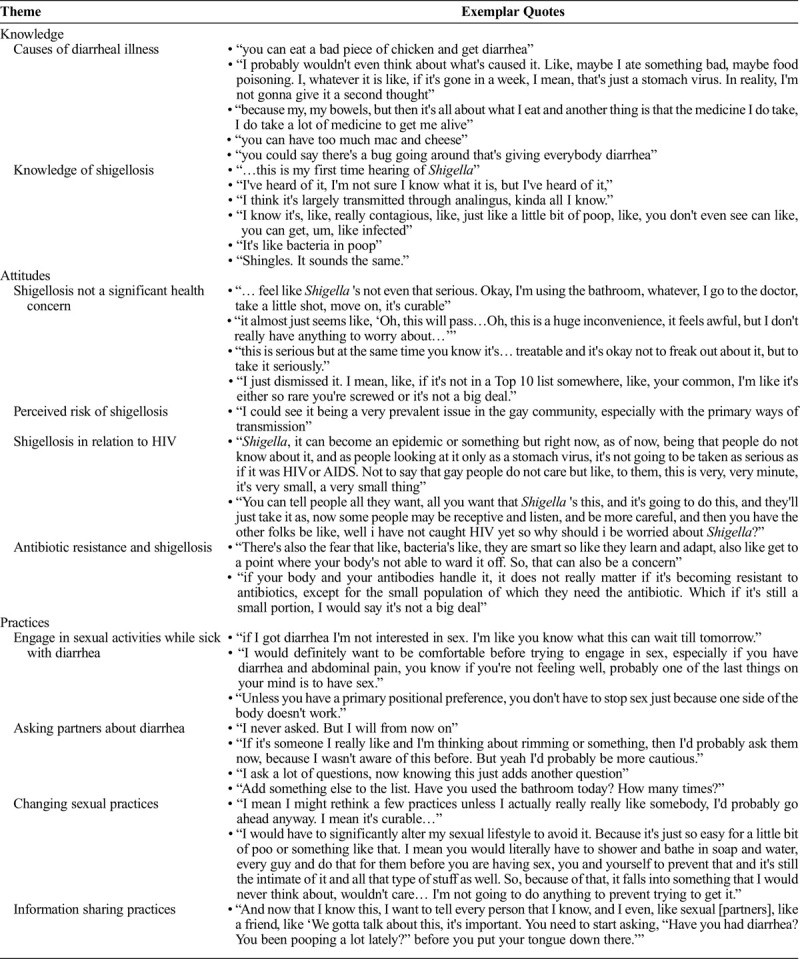
Shigellosis-Related Knowledge, Attitudes, and Practices Among Focus Group Participants and Exemplar Quotes

When asked about diarrhea more generally, participants tended to attribute symptoms of diarrhea to food poisoning, ingesting certain food items or medication, and stomach bugs (Table 2). Because of the perceived causes of diarrhea, participants did not interpret diarrheal symptoms to be a concern, with one participant indicating he “probably would not even think about what's caused it [diarrhea]. Like, maybe I ate something bad, maybe food poisoning. I, whatever it is like, if it's gone in a week, I mean, that's just a stomach virus. In reality, I'm not gonna give it a second thought.”

Although baseline levels of knowledge about *Shigella* and shigellosis were low, when presented with educational information about shigellosis, participants asked a range of questions suggesting an interest in learning more about the disease (Table 3). Participants inquired about testing and treatment, short- and long-term health outcomes, vehicles of transmission (e.g., fomites, food, and water). prevalence of shigellosis among gay and bisexual men and among people living with HIV, level of health care provider knowledge during diagnosis and treatment, and signs and symptoms of illness.

**TABLE 3:**
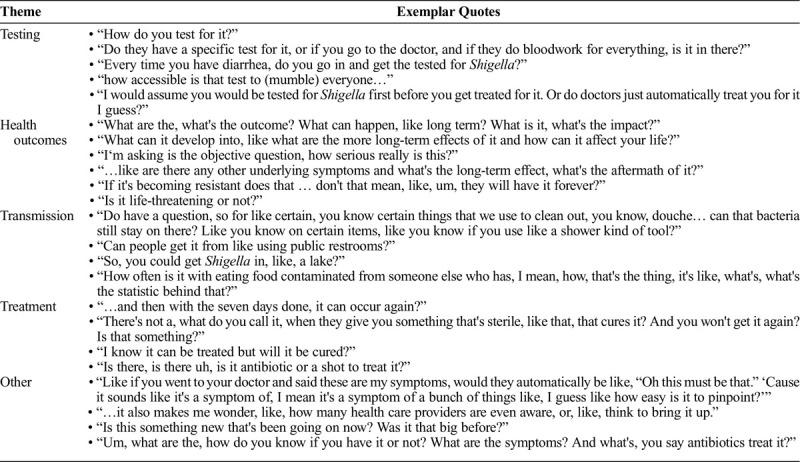
Questions Participants Had After Being Presented With General Shigellosis Information

### Attitudes

Participants across all focus groups did not perceive shigellosis to be a significant health concern (Table 3) because it is treatable, has minor symptoms, and often resolves on its own. A minority of participants believed that, because they had not heard about shigellosis before, it was likely uncommon and not a serious health concern, saying “I just dismissed it. I mean, like, if it's not in a top 10 list somewhere… I'm like it's either so rare you are screwed or it's not a big deal.” When asked about perceived risk of shigellosis, several participants felt that either they or gay and bisexual men in general were at risk of shigellosis because of sex-related risk factors for shigellosis, “I could see it being a very prevalent issue in the gay community, especially with the primary ways of transmission.”

Some participants indicated concern about the potential for *Shigella* to be antimicrobial resistant, indicating that “There's also the fear that like, bacteria's like, they're smart so like they learn and adapt, also like get to a point where your body's not able to ward it off. So, that can also be a concern.” This perception was not ubiquitous. Several participants were not concerned about antimicrobial resistance because of perceptions about the low prevalence of resistance and/or perceptions about immune system ability to fight *Shigella* bacteria: “If your body and your antibodies handle it, it doesn't really matter if it's becoming resistant to antibiotics, except for the small population of which they need the antibiotic. Which if it's still a small portion, I would say it's not a big deal.”

Several participants framed their perceptions about shigellosis in relation to HIV, indicating that, compared with HIV, shigellosis would not be perceived as a major health concern. For example, one participant described that “*Shigella*, it can become an epidemic or something but right now, as of now, being that people do not know about it, and as people looking at it only as a stomach virus, it's not going to be taken as serious as if it was HIV or AIDS. Not to say that gay people do not care but like, to them, this is very, very minute, it's very small, a very small thing.”

### Practices

Across all focus groups, most participants reported that they do not, or would not, engage in sexual activities while sick with diarrhea because “If I got diarrhea I'm not interested in sex. I'm like you know what this can wait till tomorrow.” One participant, however, indicated that he would adjust sexual behaviors if he had diarrhea, remarking that “Unless you have a primary positional preference, you don't have to stop sex just because one side of the body doesn't work.”

Participants had mixed reactions when asked if they would ask sex partners if they recently had diarrhea. Multiple participants responded with a “no,” whereas others suggested that they might ask or would consider asking partners about recent diarrhea. For example, some participants indicated that they would ask about previous diarrhea saying “if it's someone I really like and I'm thinking about rimming or something, then I'd probably ask them now, because I wasn't aware of this before. But yeah I'd probably be more cautious.” Others indicated that, because they already ask partners a range of questions about their health status, they could add asking about diarrhea to already routine behavior. One participant described that he has a list of sexual health-related questions that he asks sexual partners, and after learning about shigellosis, he will “Add something else to the list. Have you used the bathroom today? How many times?”

Overall, participants reported they would generally be unlikely to change sexual practices after learning about shigellosis. One participant explained that “I would have to significantly alter my sexual lifestyle to avoid it. Because it's just so easy for a little bit of poo or something like that. I mean you would literally have to shower and bathe in soap and water, every guy and do that for them before you're having sex… so because of that, it falls into something that I would never think about, wouldn't care … I'm not going to do anything to prevent trying to get it.” Participants indicated that because they did not perceive shigellosis to be a significant health concern, they would not be motivated to change their behaviors, saying “I mean I might rethink a few practices unless I actually really really like somebody, I'd probably go ahead anyway. I mean it's curable…” Participants also stated that they would be unwilling to abstain from sex for 2 weeks after symptoms resolve, when an individual can still be contagious. Very few participants indicated that they would change their sexual practices after learning about shigellosis, including engaging in more hygiene behaviors, avoiding sex “if things aren't looking right” or if they are experiencing symptoms of diarrhea, and asking partners about their sexual history.

## DISCUSSION

Across all focus groups, participants had limited or no knowledge of shigellosis, with most never having heard of it. These findings are consistent with other work that has demonstrated low levels of knowledge of shigellosis among MSM.^8,10,18^ In addition, when discussing diarrhea generally, most participants indicated that they attribute diarrheal illness primarily to food poisoning or food-related causes. Similar findings have been described in other studies that have found among the general population; most people perceive that individuals tend to get sick with diarrhea due to food poisoning, with only a small number perceiving that people can get diarrhea from other people.^20^ Although *Shigella* can spread through food and many foodborne outbreaks of shigellosis have been reported, outbreaks of shigellosis in the United States are primarily due to person-to-person transmission.^4^ These identified knowledge gaps highlight an opportunity for education about shigellosis as a disease and the potential for diarrheal illness to spread through nonfood transmission routes.

Upon learning about shigellosis, participants had many questions about shigellosis transmission, treatment, health outcomes, and prevalence. These results capture specific aspects of shigellosis and diarrhea that participants had limited knowledge of, and indicate an openness toward education about diarrhea causes and transmission, and shigellosis as a potential cause of diarrheal illness among gay and bisexual men. To increase knowledge of shigellosis among MSM, strategies such as health communication and social marketing could be used, and these efforts should focus on providing answers to the types of questions respondents asked about shigellosis. Health communication and social marketing have been used successfully to raise awareness and promote healthy sexual behaviors.^21–23^ Lessons from these efforts emphasize the importance of using culturally appropriate and nonstigmatizing messages and images when trying to reach MSM, and can be applied to shigellosis efforts.^24^

Participants in this study did not perceive shigellosis to be a serious health concern, especially in relation to other health concerns, particularly HIV. Participants asked multiple questions about the short- and long-term health outcomes of becoming sick with shigellosis, suggesting that the low perceived severity could be related to, or a function of, a lack of knowledge about shigellosis. Future health communication and social marketing efforts may need to include messages about the possibility of severe health outcomes related to shigellosis including bloodstream infections, reactive arthritis, and irritable bowel syndrome,^2^ and the potential for more severe illness among those with compromised immune systems.^25–27^ However, care may be needed not to overstate the potential health impact of shigellosis because these outcomes are not common. Qualitative research with MSM who have had shigellosis could identify how to frame perceptions of severity in a way that is accurate, is motivating, and captures the range of health outcomes of shigellosis.

After learning about shigellosis, participants in this study reported mixed intentions to engage in behaviors that may protect against shigellosis, including changing sexual behaviors or talking with sexual partners about diarrhea. However, none of the participants in this study were previously diagnosed with shigellosis. Therefore, it is not clear if participants' mixed intention to engage in protective behavior would translate to real-life behavior if sick with shigellosis. Future studies should be conducted among MSM who have a previous shigellosis diagnosis to supplement information on what participants in this study reported intending to do if sick.

The lack of intention to engage in protective behaviors among participants was not surprising given the low levels of knowledge and low perceived severity of shigellosis. Across multiple theories of health behavior, knowledge and perceived severity are important determinants of health behavior.^28^ The Health Belief Model posits that, in addition to other determinants, individuals must perceive that the consequences of a particular health problem are serious as a prerequisite for engaging in particular health behaviors,^29^ whereas the Integrated Theory of Health Behavior Change suggests that increased levels of knowledge influence a range of other determinants that influence preventative health behaviors.^30^ Although this study was not theory-driven and was not designed to explore relationships between constructs such as knowledge, perceived severity, and behavior, relationships between constructs in behavior change theories can help interpret the qualitative findings of this study.

Behaviors to prevent becoming sick with shigellosis are varied and include washing hands, avoiding swallowing water when swimming, and following safe food and water behaviors when traveling. In addition, among those who have shigellosis, washing hands, avoiding swimming, and not cooking for others. With regard to sex-related prevention strategies, these include using condoms and other barriers (such as dental dams) during sexual activity, washing genitals and the anus before sex, and avoiding sex while sick with shigellosis. For these sex-related prevention strategies for shigellosis, more research is needed to determine which are most effective for preventing shigellosis and which are most effective at preventing the spread of shigellosis among people who are sick or recently sick. In addition, both qualitative and quantitative research is needed to understand the determinants of both general and sex-related shigellosis prevention behaviors among MSM. Finally, theory-driven research should be conducted to identify variability in determinants of behaviors among different subpopulations of MSM who may be at particular risk of becoming sick with shigellosis (e.g., international travelers and those with multiple sex partners), of spreading shigellosis to others (e.g., those working as food handlers or in health care), or of having more severe health outcomes (e.g., those who are immunocompromised).

KAPs identified in this study, including lack of knowledge and openness to learning more about shigellosis, may be different from shigellosis-related KAPs among gay and bisexual men in other parts of the United States and may be different from MSM who do not identify as gay or bisexual. This was a small qualitative study conducted among gay and bisexual men in Atlanta, GA, and results are not generalizable to other populations. Additional qualitative and quantitative work is needed to examine shigellosis-related KAPs among a wider range of MSM in the United States and among subpopulations of MSM who may be at particular risk.

Shigellosis prevention efforts among gay, bisexual, and other MSM could include health education and communication aimed at increasing knowledge about shigellosis and shifting perceptions about the severity of shigellosis. Future research that describes effective shigellosis prevention methods among gay, bisexual, and other MSM, including strategies for promoting prevention behaviors, is needed.
